# UV/Fe(II)/S(IV) Pretreatment for Ultrafiltration of *Microcystis aeruginosa*-Laden Water: Fe(II)/Fe(III) Triggered Synergistic Oxidation and Coagulation

**DOI:** 10.3390/membranes13050463

**Published:** 2023-04-25

**Authors:** Huarong Yu, Haiyang Yang, Guangmei Wei, Naresh Mameda, Fangshu Qu, Hongwei Rong

**Affiliations:** 1 School of Civil Engineering, Guangzhou University, Guangzhou 510006, China; 2 Key Laboratory for Water Quality and Conservation of the Pearl River Delta, Guangzhou University, Guangzhou 510006, China; 3 Department of Engineering Chemistry, College of Engineering, Koneru Lakshmaiah Education Foundation, Vaddeswaram 522303, India

**Keywords:** algae-laden water treatment, UV/Fe(II)/S(IV), membrane fouling, extracellular organic matter, synergistic mechanism

## Abstract

Ultrafiltration (UF) has been proven effective in removing algae during seasonal algal blooms, but the algal cells and the metabolites can induce severe membrane fouling, which undermines the performance and stability of the UF. Ultraviolet-activated sulfite with iron (UV/Fe(II)/S(IV)) could enable an oxidation-reduction coupling circulation and exert synergistic effects of moderate oxidation and coagulation, which would be highly preferred in fouling control. For the first time, the UV/Fe(II)/S(IV) was systematically investigated as a pretreatment of UF for treating *Microcystis aeruginosa*–laden water. The results showed that the UV/Fe(II)/S(IV) pretreatment significantly improved the removal of organic matter and alleviated membrane fouling. Specifically, the organic matter removal increased by 32.1% and 66.6% with UV/Fe(II)/S(IV) pretreatment for UF of extracellular organic matter (EOM) solution and algae-laden water, respectively, while the final normalized flux increased by 12.0–29.0%, and reversible fouling was mitigated by 35.3–72.5%. The oxysulfur radicals generated in the UV/S(IV) degraded the organic matter and ruptured the algal cells, and the low-molecular-weight organic matter generated in the oxidation penetrated the UF and deteriorated the effluent. The over-oxidation did not happen in the UV/Fe(II)/S(IV) pretreatment, which may be attributed to the cyclic redox Fe(II)/Fe(III) coagulation triggered by the Fe(II). The UV-activated sulfate radicals in the UV/Fe(II)/S(IV) enabled satisfactory organic removal and fouling control without over-oxidation and effluent deterioration. The UV/Fe(II)/S(IV) promoted the aggregation of algal foulants and postponed the shift of the fouling mechanisms from standard pore blocking to cake filtration. The UV/Fe(II)/S(IV) pretreatment proved effective in enhancing the UF for algae-laden water treatment.

## 1. Introduction

Seasonal algal blooms have been widely reported in reservoirs and lakes, causing numerous adverse effects on aquatic ecosystems. Specifically, the algae metabolites cause severe odor issues and the release of hepatotoxins, threatening drinking water safety and public health [1,2]. In addition, algal blooms increase the cost of water purification and can also lead to pipeline clogging, increasing operational and maintenance costs [3]. Ultrafiltration (UF) membranes can effectively retain algal cells without impairing cellular integrity, thus avoiding the release of intracellular substances [4]. However, algal cells and extracellular organic matter (EOM) could result in severe membrane fouling, increasing energy consumption and shortening the lifespan of the membrane [5,6].

Pretreatments such as coagulation [3], adsorption [7], oxidation [8], and the combined processes have proved effective in mitigating membrane fouling caused by algal cells and extracellular organic matter (EOM). Pre-oxidation could degrade macromolecules and decrease the hydrophobicity of the organic matter, and thus retard membrane fouling. Advanced oxidation processes (AOPs) as a pretreatment for algae-associated UF fouling control are summarized in Table 1. It can be found that oxidation with strong radicals, like in UV/H_2_O_2_, UV/chlorine, ultrasonic/PAA, ozonation, and UV/S(IV), may induce algal cell lysis or degradation of high molecular weight (MW) EOM to low MW, which would aggravate membrane fouling [9,10,11,12]. Moderate oxidation with oxidants at relatively low dosages or with iron as an activator and a coagulant always resulted in better fouling alleviation [8,12,13,14,15]. Therefore, a synergy of moderate oxidation and coagulation may be optimal for controlling algae-associated fouling.

Sulfate-based (peroxodisulfate, persulfate, peroxymonosulfate, etc.) advanced AOPs are known to be green, efficient, and cost-effective [7,8,16]. Ultraviolet-activated sulfate system (UV/S) could generate sulfate radicals (SO_4_^•−^) with relatively high oxidation capacity (E_0_ = 2.65–3.1 V), effectively alleviating membrane fouling [16]. Yun et al. [7] prepared ordered mesoporous carbon materials catalyzing peroxydisulfate, which produced various radicals (SO_4_^•−^, ^•^OH, O_2_^•−^ and ^1^O_2_), substantially reduced dissolved organic carbon and UV_254_ in the effluent, and reduced reversible resistance by 59.5–83.2%. Yang et al. [8] effectively controlled the flux decline and membrane fouling by shifting the membrane fouling mechanism from dual pore blocking and cake filtration to single intermediate pore blocking using the Fe(II)/persulfate pretreatment.

The AOP based on sulfate radicals showed its superiority due to the higher oxidation potential, longer half-life, and broader applicable pH in comparison with the AOP induced by OH^•−^. The most commonly adopted persulfate-based AOP proved highly efficient, but not economical, and it may cause toxicity issues [17]. On the other hand, sulfite was much cheaper and safer. With ultraviolet radiation, sulfite could be transformed into multiple oxysulfur radicals, i.e., sulfite radical (SO_3_^•−^), peroxymonosulfate radical (SO_5_^•−^), and sulfate radical (SO_4_^•−^), as shown in Equations (S1)–(S12) in the Supplementary Information [18,19]. Sulfite-based AOP coupled with UV activation and ferrous redox could generate a very strong oxidant (sulfate radical (SO_4_^·−^)) and highly active reductant (hydrated electron (e_aq_^−^)), and the oxidant/reductant couple could react with Fe(II)/Fe(III) and enable an oxidation-reduction coupling cycle. Thus, the UV/Fe(II)/S(IV) allows an effective synergy of moderate oxidation and coagulation, which is preferred in membrane fouling control. The UV/Fe(II)/S(IV) has been proven effective in fouling control in the nanofiltration of natural organic matter and humic substances [20,21]. The moderate oxidation and coagulation synergy induced by UV/Fe(II)/S(IV) may be very suitable for algae-associated fouling control, but this has not been systematically investigated.

In this work, the performance and mechanisms of the UV/Fe(II)/S(IV) pretreatment for UF of *Microcystis aeruginosa*–containing water were investigated, and the stand-alone S(IV), UV/S(IV), and Fe(II)/S(IV) were also examined for comparison. The effects of S(IV)-based pretreatments on the removal of contaminants in algae-laden water (ALW) were investigated. The evolution of the fouling mechanism and the fouling resistance were studied. A radical quenching experiment was also performed to elucidate the free radical action in the redox.

## 2. Materials and Methods

### 2.1. Feed Water and Reagents Preparation

This study used laboratory-cultured algal solutions to simulate the algae-laden water. *Microcystis aeruginosa*, the dominant algal specie in the algae blooms, was selected as the experimental algae species [22]. BG-11 (Blue-Green Medium) medium [23] with the ingredients shown in Table S1 was used to culture *Microcystis aeruginosa*. Specifically, the medium was prepared with ultrapure water in a 1000 mL flask. The medium was sterilized at 121 °C for 20 min and cooled to room temperature. The algae were inoculated into the medium and cultured in a climatic incubator (SPX-150B, Tianjin TST Instrument Co., Ltd., Tianjin, China) at the temperature of 25 ± 0.5 °C, an intermittent light intensity of 5000 lux, and a light duration of 14 h/d. During the incubation, the *Microcystis aeruginosa* solutions were shaken periodically to distribute the algal cells and exposed to light. The optical density (OD) at 685 nm was measured to monitor the growth of algal cells. The algal cells reached the stationary phase after approximately 35 days of incubation [24]. Finally, a diluent of NaCl (15.0 mM/L), CaCl_2_ (0.5 mM/L), and NaHCO_3_ (1.0 mM/L) was prepared, and the algal solution was diluted to 2.0 × 10^8^ cells/mL as the ALW for this experiment. The dissolved organic carbon (DOC) concentration in the ALW was about 6.8 mg/L, and the UV_254_ value was 0.20 cm^−1^. To further investigate the effect of organic matter on membrane fouling, EOM was extracted from algae-containing water by centrifugation, using a high-speed freezing centrifuge (H2050R, Xiangyi, Changsha, China) with a centrifugal force of 10,000× *g* for 10 min at 4 °C. Subsequently, the supernatant was filtered with a 0.45 μm mixed cellulose filter (Taoyuan Co., Ltd., Hainig, China) to obtain the EOM solution.

### 2.2. Experimental Setup and Protocol

**Figure 1:**
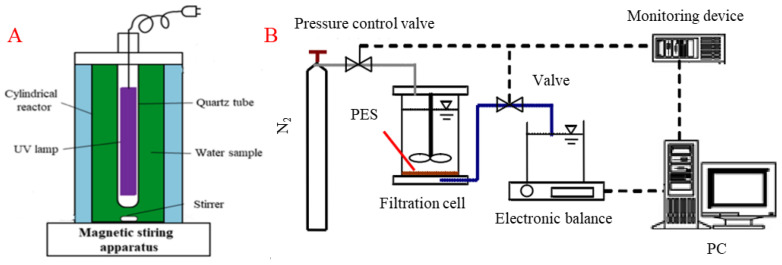
Schematic diagrams of UV radiation reactor (**A**) and UF membrane filtration experimental setup (**B**).

The experimental system consisted of a UV reactor and UF filtration system, as shown in Figure 1. UV radiation pretreatment was carried out in a reactor comprising a low-pressure UV lamp, a quartz casing, a plexiglass reactor, and a magnetic stirrer. The reactor was cylindrical, with the inner diameter, height, and adequate volume of 10 cm, 15 cm, and 1100 mL, respectively. A low-pressure mercury lamp (GPH135T5L/4, Heraeus) was selected as the UV lamp with a power of 5 W and radiation wavelength of 254 nm, arranged on the central axis of the cylindrical reactor. The UV lamp was turned on for 30 min in advance to ensure stable UV lamp output. To improve the penetration effect of UV light, the UV lamp was completely immersed in the water during the pretreatment process. The iodide-iodate chemical actinometry determined the incident light intensity, and the average fluency rate was estimated to be 1.31 mW·cm^−2^ based on the integrated form of the Beer-Lambert law [25,26]. The UV intensity was set as 2.36 × 10^3^ mJ/cm^−2^, and the irradiation was continued for 20 min to guarantee the effectiveness [27].

The UF system consisted of a filtration cell (Amicon 8400, Millipore, Burlington, MA, USA), an electronic balance (BSA2202, Saturis, Aßlar, Germany), a data recording system, and a high-pressure nitrogen cylinder (Figure 1B). The filtration volume was set to 300 mL. The UF was conducted at a constant pressure of 100 kPa. Filtration was conducted in dead-end mode, and no stirring was set during the filtration. First, the pure water flux was measured and recorded. After that, the EOM or algae solution was filtered, and the final flux was recorded. The fouled membrane was then reversed for a 2 min hydraulic backwash to remove the deposited contaminants with the pressure remaining at 100 kPa. Finally, 100 mL of pure water was filtered again, and the flux was recorded. An electronic balance (BSA2201, Sartorius, Göttingen, Germany) was connected to the computer, and the data were collected automatically every 5 s. Polyethersulfone (PES) plate UF membrane (MSC76100, Mosel, Shanghai, China) with a molecular weight cutoff (MWCO) of 100 kDa, an effective area of 45 cm^2^, a diameter of 76 mm, a contact angle between 55–60°, and a surface zeta potential of 15.58–17.04 mV was used.

Four pretreatments, i.e., stand-alone S(IV), UV/S(IV), Fe(II)/S(IV), and UV/Fe(II)/S(IV) were examined and compare with the control group. The effects of different redox systems in organic matter removal and membrane fouling performance were evaluated. S(IV) was set at 5 dosing concentrations of 0, 0.5, 1.0, 1.5, and 2.0 mM/L in all groups. The UV irradiation time and Fe dosage were set as 20 min and 0.04 mM/L, respectively. Reagents were added directly to the feed water and stirred for 2 min to mix thoroughly for subsequent filtration. Furthermore, to verify the role of free radicals in degrading contaminants and mitigating membrane fouling, methanol (MeOH) was used to quench radicals by being injected into the feed water before filtration. The MeOH dosage was set at 0.3 mM, twice the optimal S(IV) dosage. Each experimental group was performed in triplicate.

### 2.3. Membrane Fouling Evaluation

The UF membrane fouling was evaluated by flux and fouling resistance. The flux decline during ultrafiltration was described by the normalized flux (*J*/*J*_0_). The membrane fouling resistance was divided into reversible and irreversible fouling resistance, which was calculated by the resistance model of Darcy’s formula [28,29]. Specifically, the fouling that can be removed by backwash was defined as reversible fouling, while fouling that cannot be removed was considered irreversible. According to Equations (1) and (2), the fouling resistance could be evaluated via the resistance-in-series model [4,30].

(1) $$R_{r}=\frac{\Delta P}{\mu J_{1}}-\frac{\Delta P}{\mu J_{2}}$$

(2) $$R_{ir}=\frac{\Delta P}{\mu J_{2}}-\frac{\Delta P}{\mu J_{0}}$$

where *R_ir_* is the irreversible resistance (m^−1^), *R_r_* is the reversible resistance (m^−1^), Δ*P* is operating pressure (Pa), *μ* is dynamic viscosity (Pa s), *J*_0_ is the average pure water flux of pristine membrane, *J*_1_ is the permeate flux at the end of filtration ((L/m^2^•h), *J*_2_ is the average pure water flux after hydraulic backwashing.

Hermia differential formal model was applied to simulate and analyze the flux data [31,32]. The *J*/*J*_0_ data were fitted to the filtration time (*t*) by nonlinear optimization in MATLAB. A curve of *d*^2^*t*/*dV*^2^ versus *dt*/*dV* was plotted from the original filtration data and modeled fitting, and the type of membrane contamination was determined with exponent *n* (Equation (3)).

(3) $$\frac{d^{2}t}{dV^{2}}=k{\left(\frac{dt}{dV}\right)}^{n}$$

where *n* values of 0, 1, 1.5, and 2 represent cake filtration, intermediate blocking, standard blocking, and complete pore blocking, respectively.

### 2.4. Analytical Methods

Dissolved organic carbon (DOC) concentration was determined by a total organic carbon analyzer (TOC-L CPH, Shimadzu, Kyoto, Japan). The values of UV_254_ and OD_685_, representing unsaturated organic matter and algal cell count, were measured by UV/Visible photometer (UV-1800, MAPADA, Shanghai, China). Samples were prefiltered with 0.45 μm glass fiber (Taoyuan Co., Ltd.) membrane. The molecular weight (MW) distribution of the EOM solution was determined by the UF fractionation method using cellulose acetate membranes (Taoyuan Co., Ltd.) with MW cutoff of 3, 10, 30, and 100 kDa under the constant pressure of 0.1 MPa [29]. The effective area of the membrane was 45 cm^2^, and the diameter was 76 mm.

Fluorescent organic components in the permeate were measured by three-dimensional fluorescence spectra (EEM, RF6000, Shimadzu, Kyoto, Japan). All sample solutions were prediluted equally to an absorbance less than 0.05 cm^−1^ [33]. The excitation and emission wavelengths were set to 200–450 nm and 220–550 nm, with scanning intervals of 5 nm and 1 nm, respectively. The pH of the water sample was adjusted to 6.9–7.1 before analysis and, water Raman scattering was eliminated by subtracting a controlled fluorescence spectrum of pure water from all fluorescent spectra [34]. The laser particle size meter (Bettersize 2600, Dandong Baxter, Dandong, China) was applied to measure the particle size distribution of algal flocs. A scanning electron microscope (SEM, JSM-7610F Plus, Nippon Electron, Kyoto, Japan) was used to visualize the surface micromorphology of the pristine and fouled membranes. The membrane samples were dried at room temperature and pretreated by spraying gold on the samples with an ion sputter coater (JEC-3000FC, JEOL, Kyoto, Japan).

## 3. Results and Discussion

### 3.1. Characteristics of Feed Water

The diameter of algae cells ranged from 1 to 8 μm (Figure S1A). The cell aggregation due to the EOM might influence the particle size distribution measurement [29]. The MW of EOM showed a bimodal distribution, dominated by substances with small molecules (<3 kDa) and large molecules (>100 kDa) (Figure S1B). In terms of DOC, the proportion of small and large molecules was similar, 43.2% and 44.1%, respectively. The ratio of small molecular protein components was 44.0%, which was slightly higher than that of macromolecular proteins (37.7%). By contrast, the content of small-molecular polysaccharides (55.8%) was higher than that of large-molecular polysaccharides (30.6%).

### 3.2. Organic Matters Removal with Different Pretreatments

#### 3.2.1. UV_254_ and DOC in Varying Pretreatment Systems

**Figure 2:**
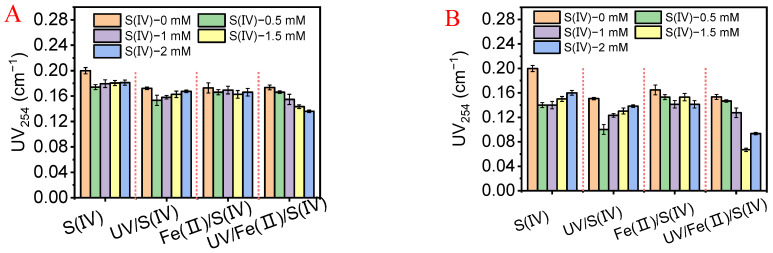
UV_254_ of the UF permeates with various pretreatments and different S(IV) concentrations for treating (**A**) EOM solution and (**B**) ALW.

Effluent UV_254_ and DOC as indicators of organic matter removal were measured in various pretreatment systems. Figure 2A shows the UV254 of the UF effluent for different pretreatment options and at different S(IV) concentrations. As shown in Figure 2A, the UV_254_ of the permeate of UF without any pretreatment was 0.200 ± 0.005 cm^−1^. The S(IV) alone at 0.5 mM allowed for much lower UV_254_, but a further increase in the S(IV) dosage did not significantly improve the UV_254_ removal. In the UV/S(IV) system, the UV_254_ removal increased first and then decreased, reaching the lowest UV_254_ value (0.1534 ± 0.008 cm^−1^) at S(IV) of 0.5 mM. Under the UV-activated S(IV) strategy, high MW organic matter could be degraded to low MW substances [35,36]. It can be speculated that higher S(IV) dosages promoted the generation of typical radicals (SO_4_^•−^) and subsequently decomposed the macromolecules [37,38]. By contrast, Fe(II)/S(IV) groups showed approximately 15% removal of UV_254_. The Fe(II) may have acted as a coagulant, and coagulation and membrane separation synergistically improved the removal of organic matter [1]. For UV/Fe(II)/S(IV) system, the removal efficiency of UV_254_ showed a noticeable increase as the S(IV) dosage increased, and the effluent UV_254_ reduced to 0.1358 ± 0.002 cm^−1^ at the S(IV) dosage of 2.0 mM.

It is worth noting that SO_4_^•−^ may also react with Fe(II) to form in situ Fe(III) [1]. The in situ Fe(III) may facilitate highly efficient coagulation [31]. Hence, free radical oxidation and coagulation may synergistically enhance the subsequent UF. The coupling of oxidation and coagulation in the UV/SO_3_^2−^/Fe(III) system has also been reported [39]. The removal of DOC in each group (Figure S2A in SI) was similar to the decrease in UV_254_. In the stand-alone S(IV) group, the removal rate progressively increased as S(IV) increased from 0 mM to 2.0 mM. Nevertheless, the DOC removal in the S(IV) group was still much less than that in the UV/S(IV), Fe(II)/S(IV), and UV/Fe(II)/S(IV) groups.

Figure 2B shows the UV_254_ in the permeate of UF with various pretreatments for treating ALW. The UV_254_ of the UV/S(IV)-UF effluent decreased first and then gradually increased with the increasing S(IV) dosage. The excess free radicals generated by UV radiation S(IV) could cause algae cell lysis and the release of intracellular substances, thus deteriorating the permeate quality. The removal of UV_254_ by the Fe(II)/S(IV)-UF fluctuated, indicating the mildness of the Fe(II) coagulation. As expected, UV/Fe(II)/S(IV) system showed the best UV_254_ removal. In terms of DOC removal, the UV/S(IV), Fe(II)/S(IV), and UV/Fe(II)/S(IV) groups showed better DOC removal than the stand-alone S(IV) group (Figure S2B in SI). The organic removal values in the tests with ALW were always higher than those with EOM, which indicated that the algal cells would assist in organics removal in UF, possibly through adsorption or coagulation [40]. Furthermore, excessive radicals that may lead to algal cell lysis should be avoided in the pretreatment [1].

#### 3.2.2. The Removal of Fluorescent Organics with Different Pretreatments

**Figure 3:**
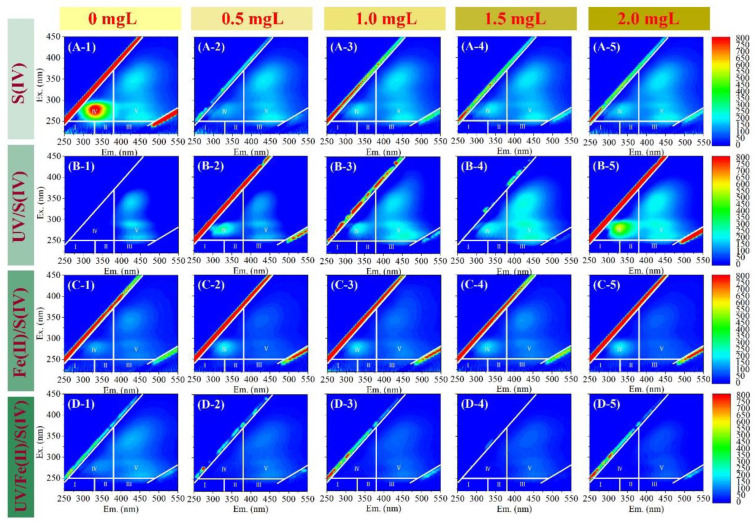
EEM of the permeate of UF treating EOM solution with different pretreatments and S(IV) dosages. (**A-1**–**A-5**) S(IV), (**B-1**–**B-5**) UV/S(IV), (**C-1**–**C-5**) Fe(II)/S(IV), and (**D-1**–**D-5**) UV/Fe(II)/S(IV) pretreatment systems with S(IV) dosage at 0, 0.5, 1.0, 1.5, and 2.0 mg/L, respectively. UV irradiation time was 20 min, and Fe(II) dosage was 0.04 mM.

The EEM spectra of the permeate of EOM solution treated with various pretreatments are shown in Figure 3. In Figure 3(A-1), the UF permeate without pretreatment showed strong fluorescence in region IV pointing to dissolved microbial metabolites [5]. In addition, the protein-like (region I and II), fulvic acid-like (region III), and humic-like (region V) substances could also be found in the EEM spectra, which was consistent with the previous study [41]. In the stand-alone S(IV) system, 0.5 mg/L S(IV) allowed for a clear removal of protein-like substances, but S(IV) at a much higher dose did not significantly alter the fluorescence spectra of the UF permeate. In UV/S(IV) system, only humic-like substances could be found at low S(IV) dosages, while protein-like substances gradually appeared with increasing S(IV) dosages. The fluorescence of both humic-like and protein-like substances increased significantly at high S(IV) dosages. UV/S(IV) may degrade high MW biopolymers or humic substances into low MW substances that could penetrate the membrane [36]. Fluorescence of both humic-like and protein-like substances could be observed in the permeate of the UV/Fe(II)/S(IV)-UF, but the S(IV) dosage did not significantly alter the fluorescence.

**Figure 4:**
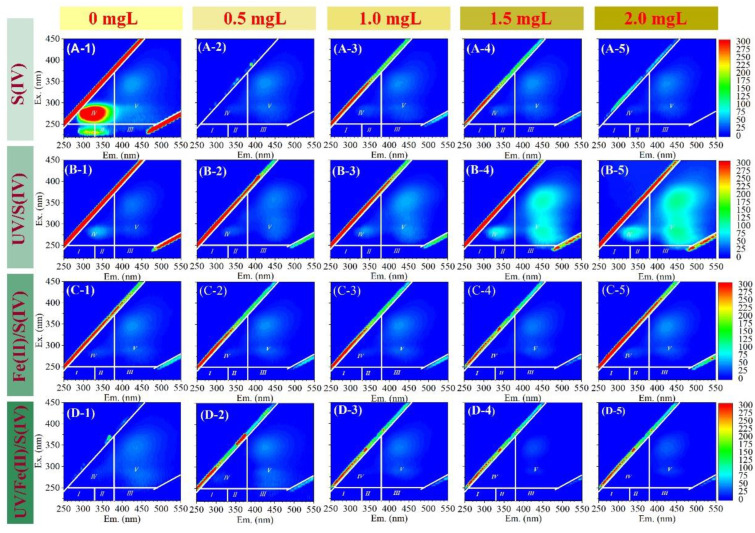
EEM of the permeate of UF treating ALW with different pretreatments and different S(IV) dosages. (**A-1**–**A-5**) S(IV), (**B-1**–**B-5**) UV/S(IV), (**C-1**–**C-5**) Fe(II)/S(IV), and (**D-1**–**D-5**) UV/Fe(II)/S(IV) pretreatment systems with S(IV) dosage at 0, 0.5, 1.0, 1.5, and 2.0 mg/L, respectively. UV irradiation time was 20 min, and Fe(II) dosage was 0.04 mM.

The EEM spectra of the permeate for treating ALW with various pretreatments can be observed in Figure 4. The UF permeate without pretreatment for ALW treatment (Figure 4(A-1)) showed much stronger fluorescence than that for EOM treatment (Figure 3(A-1)). The S(IV) alone could improve the removal of fluorescent organic matter; a further increase in S(IV) concentration did not change the fluorescence removal, which was consistence with the UV_254_ and DOC results. In the UV/S(IV) system, a low dosage of S(IV) (0.5 mM) showed the best removal of fluorescent organic substances. Continuously increasing S(IV) dosage up to 2.0 mM may generate more free radicals, which can result in algal cell rupture, and consequently, the ascending fluorescence in the permeate. Fe(II)/S(IV) system effectively removed protein-like substances, which could possibly be attributed to the synergistic effect of membrane retention and coagulation. The Fe(II)/UV/S(IV) system showed the best removal of fluorescence, which may be ascribed to the coupling effects of oxidation, coagulation, and membrane separation.

### 3.3. Membrane Fouling with Different Pretreatments

#### 3.3.1. Flux and Fouling Resistance

**Figure 5:**
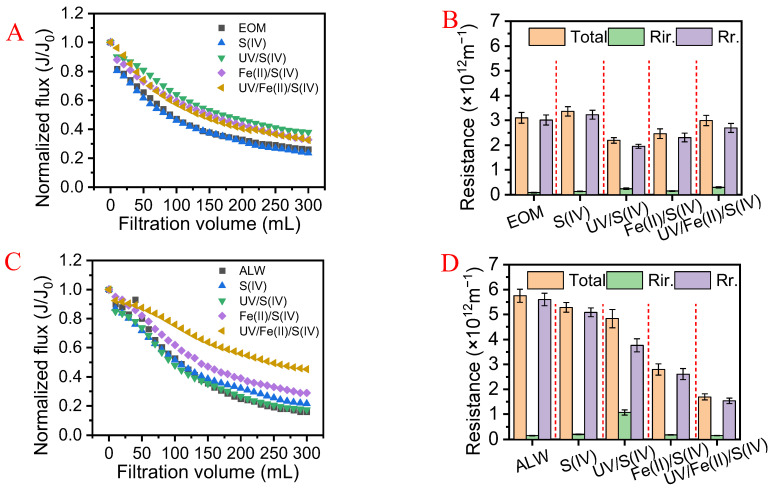
Membrane flux variation during filtration of (**A**) EOM, (**C**) ALW, and membrane fouling resistances during (**B**) EOM and (**D**) ALW. The Fe(II) concentration in the Fe(II)/S(IV) system and UV/ Fe(II)/S(IV) system was 0.04 mM, and the S(IV) concentration in the S(IV) alone, the Fe(II)/S(IV) and UV/ Fe(II)/S(IV) was 1.5 mM. UV radiation lasted for 20 min in the UV/S(IV) and UV/Fe(II)/S(IV).

Figure 5 shows the membrane flux and fouling resistance in the UF tests with different pretreatments for treating EOM and ALW. As illustrated in Figure 5A, in the UF of the EOM, a rapid drop in membrane flux was observed and the final normalized flux decreased to 0.26. Stand-alone S(IV) pretreatment did not alter the flux decline. In comparison, other pretreatments alleviated the flux decline to some extent. Particularly, UV/S(IV), Fe(II)/S(IV), and UV/Fe(II)/S(IV) systems corresponded to final normalized fluxes of 0.38, 0.33, and 0.32, respectively. The UV-activated S(IV) showed the best fouling control performance, which was consistent with previous studies [21,27]. However, the extra organic matter may access the effluent of UV/S(IV)—UF, as evidenced by the results of EEM (Figure 3(B-1–B-5)).

As shown in Figure 5C, the flux decline in the UF for treating ALW showed a different trend compared with the UF for treating EOM. The flux decline was more severe in treating ALW with the final normalized flux of 0.16. Different from EOM, the UV/S(IV) system mitigated the flux decline only slightly, with a final normalized flux of 0.18. With the presence of Fe(II), the Fe(II)/S(IV) and UV/Fe(II)/S(IV) pretreatments alleviated the flux decline significantly, corresponding to final normalized fluxes of 0.29 and 0.45, respectively. These were two to three times as high as that in the UF of raw ALW.

As illustrated in Figure 5B, when filtering the EOM, the reversible resistance reached 3.02 × 10^12^ m^−1^, accounting for 96% of the total fouling resistance (3.10 × 10^12^ m^−1^). Likewise, the stand-alone S(IV) did not alter the fouling reversibility either. UV/Fe(II)/S(IV) system mildly decreased the reversible fouling to 2.70 × 10^12^ m^−1^, while the irreversible fouling increased, resulting in almost unaltered total fouling resistance. UV/S(IV) and Fe(II)/S(IV) allowed for low reversible resistances of 1.95 × 10^12^ m^−1^ and 2.31 × 10^12^ m^−1^, respectively. In the UV/S(IV) and Fe(II)/S(IV), the irreversible resistance increased moderately to 0.25 × 10^12^ m^−1^ and 0.15 × 10^12^ m^−1^, accounting for 10.9% and 6.1% of the total fouling resistance. It is noteworthy that the irreversible fouling resistance increased in all UF with pretreatments. It has been reported that free radicals may shift the hydrophobic macromolecules into hydrophilic micromolecules while weakening the electrostatic repulsion at the membrane interface, making it easier to block membrane pores [8,21,42]. Accordingly, the increase in irreversible fouling observed in the UF with various pretreatments may be ascribed to free radicals generated in the pretreatments. At the same time, Fe(II)/Fe(III) did not exert a significant influence on the fouling in the UF tests with EOM.

In the ALW treatment (Figure 5D), the total resistance in the UF of ALW was 5.75 × 10^12^ m^−1^, of which 97% was reversible resistance and irreversible resistance was only 0.15 × 10^12^ m^−1^. Notably, membrane fouling was greatly alleviated with different pretreatments. Stand-alone S(IV) exhibited mild membrane fouling mitigation, with a high percentage of reversible fouling of 96%. In Fe(II)/S(IV) and UV/Fe(II)/S(IV) groups, the total fouling resistance was reduced to 2.79 × 10^12^ m^−1^ and 1.69 × 10^12^ m^−1^, respectively, which indicated that coagulation may be quite effective in fouling control for the algae-laden water treatment. It is of interest that there was no significant difference in irreversible resistance between Fe(II)/S(IV) and S(IV) systems, suggesting that the coagulation may have majorly altered the reversible fouling, possibly by changing the structure of the cake layer [43]. In previous studies, the in situ–formed Fe(III) integrated with oxidation was reported to effectively alleviate membrane fouling by promoting the aggregation of the algal foulants through coagulation [31]. This was also observed in the stand-alone Fe(II) system, where the trend of flux reduction was significantly alleviated with increasing Fe(II) dosage (Figure S5C).

#### 3.3.2. Fouling Mechanism Analysis

**Figure 6:**
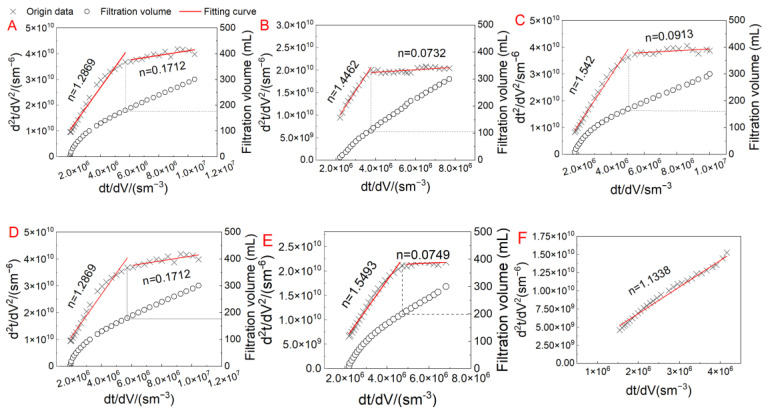

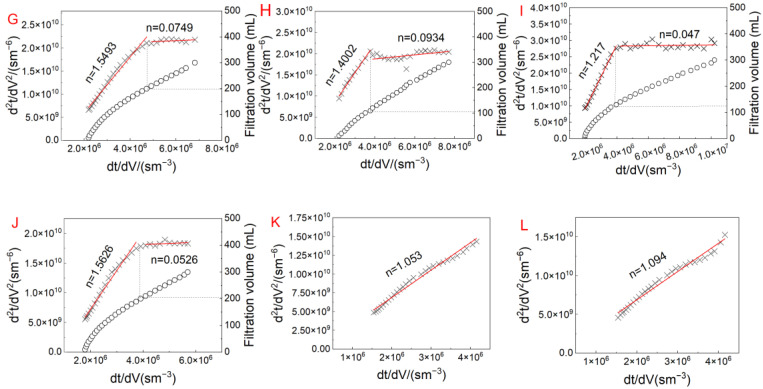
Fitting curves for the filtration data in the UV/Fe(II)/S(IV) system with different S(IV) dosages for treating EOM: (**A**) feed water, (**B**) 0 mM, (**C**) 0.5 mM, (**D**) 1.0 mM, (**E**) 1.5 mM, (**F**) 2.0 mM; and for treating ALW: (**G**) feed water, (**H**) 0 mM, (**I**) 0.5 mM, (**J**) 1.0 mM, (**K**) 1.5 mM, (**L**) 2.0 mM.

The membrane fouling mechanism was analyzed by fitting the flux data to the classic Hermia model. The *dt*/*dV* and *d*^2^*t*/*dV*^2^ relationship curves of the UV/Fe(II)/S(IV) system during the filtration of EOM and ALW at different S(IV) dosages are presented in Figure 6. The *n*-values fitted to the data were bipartite in distribution when the S(IV) dosage did not exceed 1.5 mg/L, indicating the coexistence of multiple mechanisms in the EOM filtration with UV/Fe(II)/S(IV) pretreatment. In Figure 6A–E, the *n*-values fluctuated between 1.2 and 1.6 in the first stage, suggesting that the standard pore blocking played a significant role. The small organic molecules in EOM could enter the membrane pores at the early stage, resulting in a rapid flux decline. The successive pore blocking and cake filtration were typical membrane fouling mechanisms presented in the filtration of NOM and an algal organic matter [44,45]. At the beginning of filtration, biopolymers could access the open membrane pores rapidly, leading to pore blocking, and then the organic matter gradually accumulated and formed a cake/gel layer on the membrane surface [46]. It could be found that with the increase of S(IV) dosage, the appearance of cake filtration was gradually postponed, and it disappeared (with standard pore blocking dominating the whole UF) when S(IV) increased to 2.0 mM.

When treating ALW, fouling mechanisms were dominated by standard pore blocking and cake filtration successively without the pretreatment and the UV/Fe(II)/S(IV) with low S(IV) dosages (0, 0.5, and 1.0 mM). The cake layer filtration was delayed as the S(IV) dosage increased. When S(IV) dosages increased to 1.5 and 2.0 mM, pore blocking governed the whole filtration, which may be attributed to large agglomerates formed by in situ–generated Fe(III) and the algal cells. The big agglomerates might form a loose and porous cake layer with low filtration resistance, which was not conducive to retaining small molecules [8]. The small molecules might penetrate the cake layer and continuously block the membrane pores. Meanwhile, coagulation weakened the electrostatic repulsion between the foulants and the membrane surface, strengthening the adsorption of small molecules in the membrane pores, and it tended to enhance the pore blocking [4]. A previous study also reported the transition of the membrane fouling mechanism from standard pore blocking to cake filtration with UV/S pretreatment [10].

#### 3.3.3. Surface Morphology of the Pristine and Fouled Membranes

**Figure 7:**
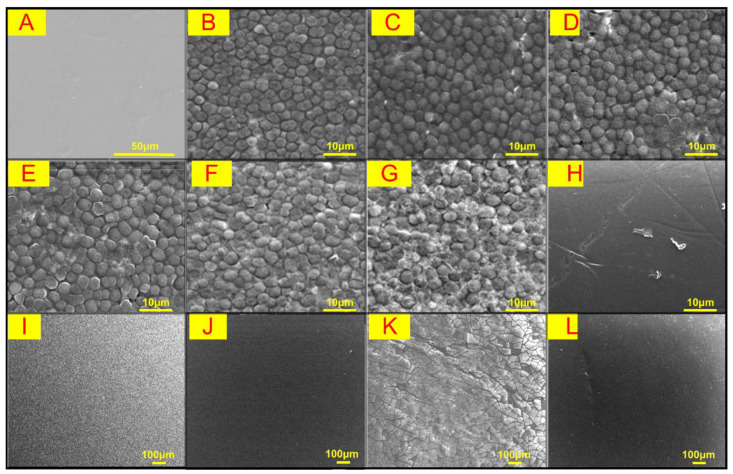
SEM images of (**A**) pristine membrane; (**B**) membrane fouled with ALW; (**C**–**G**) membrane fouled with ALW pretreated by UV/Fe(II)/S(IV) with the S(IV) dosage at 0 mM, 0.5 mM, 1.0 mM, 1.5 mM, and 2.0 mM; (**H**) membrane after backwashing; (**I**,**J**) membrane fouled with EOM and after backwashing; and (**K**,**L**) membrane fouled with EOM pretreated by UV/Fe(II)/S(IV) with the S(IV) dosage at 1.5 mM and after backwashing.

As shown in Figure 7, the pristine membrane surface was relatively smooth and flat (Figure 7A), and after filtering ALW, it was covered by dense and ordered algal cells (Figure 7B). The UV/Fe(II)/S(IV) pretreatment significantly changed the morphology of foulants on the membrane surface (Figure 7B–G). At low S(IV) dosages (<1.5 mM), algal cells tended to aggregate into clumps and form a more loose and thin fouling layer under the coagulation effect of in situ Fe(III). The deposited algal cells could be effectively removed by backwashing, while the small amount of residue after backwashing might be the organic matter that adhered to the membrane surface (Figure 7H). The membrane morphology at the S(IV) dosage of 0 mM was the same as that of ALW direct filtration, indicating the critical role of S(IV) in ensuring a strong synergistic effect of redox and coagulation. As the S(IV) dosage increased to 2.0 mM, the shrunken algal cells were observed, which may be attributed to the strong coagulation or the cell lysis. In the UF of EOM, organic matter was uniformly and densely deposited on the membrane surface. Compared with ALW, more foulants remained on the membrane surface after backwashing, indicating that EOM fouling could be more irreversible. In Fe(II)/UV/S(IV)-1.5 mM group, a slabbing of the fouling layer with a sizeable fragmented distribution was observed. After backwashing, the scattering of foulants on the membrane could be observed, which was consistent with the increased irreversible fouling (Figure 5B).

### 3.4. Role of Free Radicals and Mechanisms Decipher

#### 3.4.1. Role of Free Radicals on the Organics Removal and Fouling Alleviation

**Figure 8:**
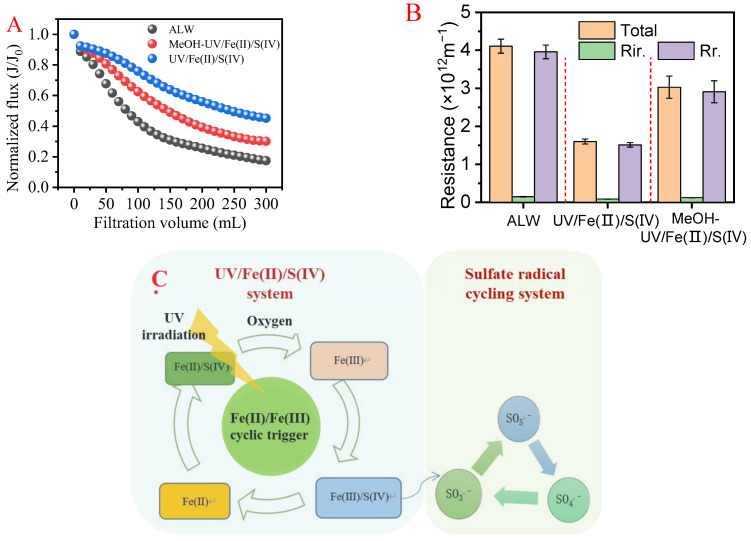
(**A**) Membrane flux variation and (**B**) Fouling resistance during ALW filtration under free radical scavenger. (**C**) The mechanism for the enhanced performance of the synergistic solid process of Fe(II)/UV/S(IV).

According to the results above, Fe(II)/UV/S(IV)-1.5 mM showed the best organic removal and fouling alleviation. The free radical scavenger (methanol) was used to elucidate the contribution of free radicals in the Fe(II)/UV/S(IV) reaction, and UF tests were also carried out. As shown in Figure 8A,B, a more severe flux decline was obtained with the scavenger than without the scavenger, while the UF of ALW without UV/Fe(II)/S(IV) treatment showed the most rapid flux decline. The flux decline ratios in UF without the scavenger, with the scavenger, and UF of raw ALW were 47%, 62%, and 72%, respectively. It can be speculated that the contribution of in situ Fe(III) coagulation and of free radicals to the flux improvement was around 10% and 15%, respectively. In terms of membrane fouling, the total fouling resistance in the group with the scavenger (3.3 × 10^12^ m^−1^) was about twice as high as in the group without the scavenger (1.6 × 10^12^ m^−1^), but it was lower than the fouling resistance in UF of raw ALW (4.1 × 10^12^ m^−1^). It could be estimated that free radicals may have contributed to about 41% of the fouling alleviation, which was two times as much as the coagulation. It is considered that the sulfate radicals substantially contribute to the organic matter removal and membrane fouling mitigation in the UV/Fe(II)/S(IV) system. Relevant studies have also reported the contribution of free radicals to the membrane fouling mitigation in UF of NOM and algal organics [35,43].

#### 3.4.2. Mechanisms 

According to the organic matter removal and the membrane fouling results, it can be inferred that the synergy of coagulation with in situ–generated Fe(III) and oxidation with sulfate radicals played an important role in UV/Fe(II)/S(IV). Specifically, under UV radiation, S(IV) could be rapidly converted to ${\mathrm{SO}}_{3}^{•-}$ and intermediate coexistence products (e_aq_^−^), including H^+^, HSO_3_^−^, and H_2_O (Equation (4)). Fe(II) combined with intermediate HSO_3_^−^ to form FeHSO_3_^+^, which further reacted with oxygen or Fe(III) to generate FeSO_3_^+^ (Equations (5)–(7)). Subsequently, in the presence of UV and oxygen, FeSO_3_^+^ could be converted into polymorphic sulfate radicals (${\mathrm{SO}}_{3}^{•-}$, ${\mathrm{SO}}_{4}^{•-}$, and ${\mathrm{SO}}_{5}^{•-}$) (Equations (8)–(12)). In this process, Fe(III) could be reverted to the reduced form of Fe(II) by a single electron transfer with HSO_3_^−^ to generate ${\mathrm{SO}}_{3}^{•-}$. Meanwhile, Fe(II) facilitated the formation of ${\mathrm{SO}}_{4}^{•-}$ and oxidation state of Fe(III) by providing a single electron to HSO_5_^−^. The redox process of Fe(II)/Fe(III) was the key trigger for the entire pretreatment system, as well as the catalyst for the redox process of S(IV) and the initiator of polymorphic sulfate radicals. The overall generation efficiency of sulfite radicals mainly depended on the redox cycling rate of Fe(II)/Fe(III). The strong oxidizing HSO_5_^−^ and the strong reducing intermediate (e_aq_^−^) produced under UV activation guaranteed the smooth oxidation-reduction coupling process [47]. In addition, in situ–generated Fe(III) could coagulate organic matter and algal cells to form particulate flocs through adsorption bridging, net sweeping, and changes in the zeta potential of algal cells [1,31]. Compared with the dense cake layer formed by direct filtration of EOM and ALW, coagulation conferred a relatively loose and porous fouling layer. The shift from standard pore blocking to cake filtration was delayed, leading to elevated final membrane flux and reduced membrane fouling. The proposed mechanism of UV/Fe(II)/S(IV)-enhanced UF is illustrated in Figure 8, and the principal reactions in the synergistic process are shown in Table 2. Figure 8C shows the two oxidant-reductant cycles of Fe(II)/Fe(III) and oxysulfur radicals. This coupling dual circulation allows for efficient and sustainable synergy of sulfate radical oxidation and coagulation. The ferrous reduction weakened the strong sulfate radical, and allowed for a moderated oxidation that effectively removed the organic matter without algal cell lysis. The iron generated in the circulation effected coagulation, which further removed organic foulants and aggregated all foulants. This again retarded membrane fouling. Therefore, coupling dual circulation shown in Figure 8C enabled effective fouling control and organic removal.

The UV/Fe(II)/S(IV) proposed in this work has proved effective in fouling control and organic removal for algae-laden water treatment. Furthermore, it is much cheaper and safer than other sulfate-radical-based AOPs, and it could be easily implemented in a regular coagulation-sedimentation tank. Therefore, it is expected that this UV/Fe(II)/S(IV) may hold promise in emergency treatment during the algae bloom.

## 4. Conclusions

In this work, UV/Fe(II)/S(IV) pretreatment was proposed to enhance the UF of algae-laden water and to alleviate membrane fouling. The following conclusions could be drawn from this study:

1. The UV/Fe(II)/S(IV)-UF showed the highest removal of organic matter, in comparison with other pretreatments, i.e., stand-alone S(IV), Fe(II)/S(IV), and UV/S(IV).
2. The UV/Fe(II)/S(IV) effectively alleviated flux decline and reduced fouling resistance. At optimal conditions, final normalized fluxes in UF of ALW and EOM increased by 12.0% and 29.0%, and reversible fouling resistance decreased by 35.3% and 72.5%, respectively.
3. The transition of the fouling mechanism from pore blocking to cake filtration was found in the UF of EOM and ALW. The UV/Fe(II)/S(IV) pretreatment delayed the transition point of the fouling mechanisms and even eliminated the cake filtration at a high S(IV) concentration.
4. In the UV/Fe(II)/S(IV) system, UV-activated S(IV) generated oxysulfur radicals, which interacted with the reductive Fe(II) and enabled redox self-cycling. The oxysulfur radicals and in situ Fe(III) exerted a synergy effect of oxidation and coagulation, which allowed efficient organic removal and alleviated membrane fouling.

## Figures and Tables

**Table 1:** AOPs pretreatment for algae-associated UF fouling control.

AOPs	Feed Water	Results	Reference
Fe(II)/PMS UV/PMS UV/Fe(II)/PMS	UF of algal EOM	DOC removal and fouling control: UV/Fe(II)/PMS > Fe(II)/PMS > UV/PMS	[13]
UV/H_2_O_2_ Coagulation	UF of algal EOM	Both reduced fouling due to the removal/breakdown of high MW substances. UV/H_2_O_2_ resulted in greater irreversible fouling due to the low MW substances generated.	[9]
Fe(II)/permanganate Fe(II)/persulfate	UF of algal EOM	Fouling control: Fe(II)/persulfate > Fe(II)/permanganate Simultaneous oxidation and coagulation alleviated fouling.	[8]
OMCs/PDS	UF of algal EOM	Reversible resistance was reduced by 59.5–83.2%, and irreversible resistance declined by 71.7–73.0%.	[7]
UV/H_2_O_2_ UV/chlorine UV/persulfate	UF of algae-laden water	Fouling control: UV/persulfate > UV/H_2_O_2_ > UV/chlorine UV/chlorine aggravated the pore-blocking fouling.	[10]
Fe(II)/S(IV)	UF of algae-laden water	Fouling was alleviated and the algal cell was intact.	[14]
PAA UV/PAA Ultrasonic/PAA	UF of algae-laden water	Fouling control: UV/PAA > PAA > Ultrasonic/PAA High dosage of PAA (> 10 mg/L) and ultrasonic led to algal cell rupture.	[11]
Fe(VI)/SPC	UF of algae-laden water	Fouling was alleviated and the algal cell was intact. Coagulation and oxidation simultaneously alleviated fouling.	[15]
Fe(II)/PS Ozone	UF of algae-laden water	Fe(II)/PS alleviated fouling. Ozonation led to algal cell lysis and thus exacerbated the fouling.	[12]

**Table 2:** Principal reactions in the UV/Fe(II)/S(IV) system [31,47].

Reaction	Equation
$SO_{3}^{2-}\;\overset{hv}{\to }\;SO_{3}^{•-}+e_{aq}^{-}$	(4)
$Fe^{2+}+HSO_{3}^{-}\to FeHSO_{3}^{+}$	(5)
$4FeHSO_{3}^{+}+O_{2}\to 4FeSO_{3}^{+}+2H_{2}O$	(6)
$Fe^{3+}+HSO_{3}^{-}\to FeSO_{3}^{+}+H^{+}$	(7)
$FeSO_{3}^{+}\;\overset{hv}{\to }\;Fe^{2+}+SO_{3}^{•-}$	(8)
$SO_{3}^{•-}+O_{2}\to SO_{5}^{•-}$	(9)
$SO_{5}^{•-}+HSO_{3}^{-}\to SO_{4}^{•-}+SO_{4}^{2-}+H^{+}$	(10)
$SO_{5}^{•-}+SO_{5}^{•-}\to 2SO_{4}^{•-}+O_{2}$	(11)
$SO_{5}^{•-}+HSO_{3}^{-}\to SO_{3}^{•-}+HSO_{5}^{-}$	(12)
$Fe^{2+}+HSO_{5}^{-}\to SO_{4}^{•-}+Fe^{3+}+OH^{-}$	(13)

## Data Availability

Data are available on demand to the authors.

## Supplementary Materials

The following supporting information can be downloaded at: https://www.mdpi.com/article/10.3390/membranes13050463/s1, Figure S1: Size distributions of Microcystis aeruginosa and molecular weight distribution of the EOM sample; Figure S2: Permeate water DOC in varying systems treating (A) EOM solution and (B) ALW; Figure S3: Membrane flux decline with varying S(IV) dosages in different pretreatments treating EOM (A) stand-alone S(IV) ALW, (B) UV/S(IV), (C) stand-alone Fe(II), (D) Fe(II)/S(IV) and (E) UV/Fe(II)/S(IV); Figure S4: Membrane fouling resistance with varying S(IV) dosages in different systems treating EOM (A) stand-alone S(IV) ALW, (B) UV/S(IV), (C) stand-alone Fe(II), (D) Fe(II)/S(IV) and (E) UV/Fe(II)/S(IV); Figure S5: Membrane flux variation with varying S(IV) dosages in different systems treating ALW (A) stand-alone S(IV) ALW, (B) UV/S(IV), (C) stand-alone Fe(II), (D) Fe(II)/S(IV) and (E) UV/Fe(II)/S(IV); Figure S6: Membrane flux variation with varying S(IV) dosages in different systems treating ALW (A) stand-alone S(IV) ALW, (B) UV/S(IV), (C) stand-alone Fe(II), (D) Fe(II)/S(IV) and (E) UV/Fe(II)/S(IV); Table S1: Ingredients of BG-11 medium.
