# Supplementary material for: UV/Fe(II)/S(IV) Pretreatment for Ultrafiltration of Microcystis aeruginosa-Laden Water: Fe(II)/Fe(III) Triggered Synergistic Oxidation and Coagulation
Source: Membranes (Basel). 2023 Apr 25;13(5):463. doi: 10.3390/membranes13050463 (PMC10222291; doi:10.3390/membranes13050463)
Supplement: Supplementary file 1 [file membranes-13-00463-s001.zip › membranes-2308488-supplementary.pdf]

Reactive species generated in the UV radiation of sulfite

$SO_3^{2-} \xrightarrow{hv} SO_3^{\cdot-} + e_{aq}^-$	k = 1.9×10^{10} M^{-1}s^{-1}	(S1)
$e_{aq}^- + O_2 \rightarrow O_2^{\cdot-}$	k = 1.9×10^{10} M^{-1}s^{-1}	(S2)
$O_2^{\cdot-} + H^+ \rightarrow HO_2$	pK = 4.8	(S3)
$O_2^{\cdot-} + HO_2 \rightarrow HO_2^{\cdot-} + O_2$	k = 9.7×10^{8} M^{-1}s^{-1}	(S4)
$HO_2^{\cdot-} + H^+ \rightarrow H_2O_2$	pK = 11.6	(S5)
$SO_3^{\cdot-} + O_2 \rightarrow SO_5^{\cdot-}$	k = 1.2×10^{9} M^{-1}s^{-1}	(S6)
$SO_5^{\cdot-} + SO_3^{2-} \rightarrow SO_3^{\cdot-} + SO_5^{2-}$	k ≈ 1.3×10^{7} M^{-1}s^{-1}	(S7)
$SO_5^{\cdot-} + SO_3^{2-} \rightarrow SO_4^{\cdot-} + SO_4^{2-}$		(S8)
$SO_4^{\cdot-} + SO_3^{2-} \rightarrow SO_3^{\cdot-} + SO_4^{2-}$	k ≈ 5×10^{8} M^{-1}s^{-1}	(S9)
$SO_5^{\cdot-} + O_2^{\cdot-} \rightarrow SO_5^{2-} + O_2$		(S10)
$SO_5^{\cdot-} + SO_5^{\cdot-} \rightarrow 2SO_4^{\cdot-} + O_2$	k ≈ 6×10^{8} M^{-1}s^{-1}	(S11)
$SO_5^{\cdot-} + SO_5^{\cdot-} \rightarrow S_2O_8^{2-} + O_2$	k ≈ 1.4×10^{8} M^{-1}s^{-1}	(S12)

Algal cell culture medium composition

Table S1 Ingredients of BG-11 medium.

Reagent	Concentration (mg/L)	Reagent	Concentration (mg/L)
$NaNO_3$	1500	$MnCl_2 \cdot 4H_2O$	1.86
K_2HPO_4	40	$Na_2MoO_4 \cdot 2H_2O$	0.22
$MgSO_4 \cdot 7H_2O$	75	$CuSO_4 \cdot 5H_2O$	0.08
$CaCl_2 \cdot 2H_2O$	36	$Co(NO_3)_2 \cdot 6H_2O$	0.05
$C_6H_8O_7$	6	Na_2CO_3	20
$C_6H_8FeNO_7$	6	H_3BO_3	2.86
$EDTANa_2$	1		

Characteristics of feed water

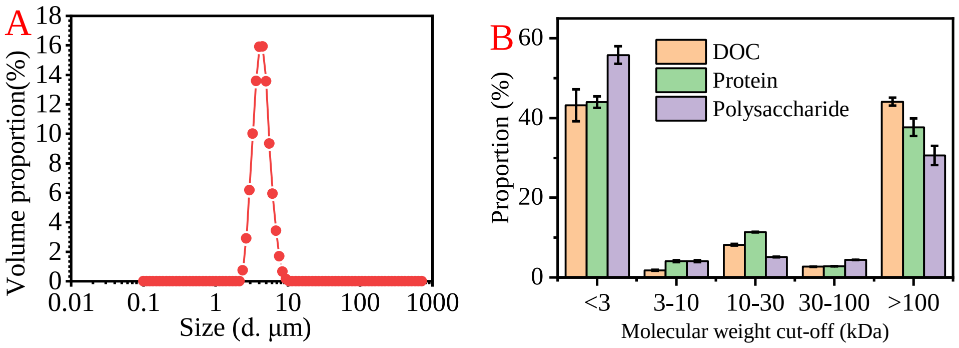

Figure S1. (A) Size distributions of *Microcystis aeruginosa*, (B) Molecular weight distribution of the EOM sample

DOC of UF permeate with different pretreatment

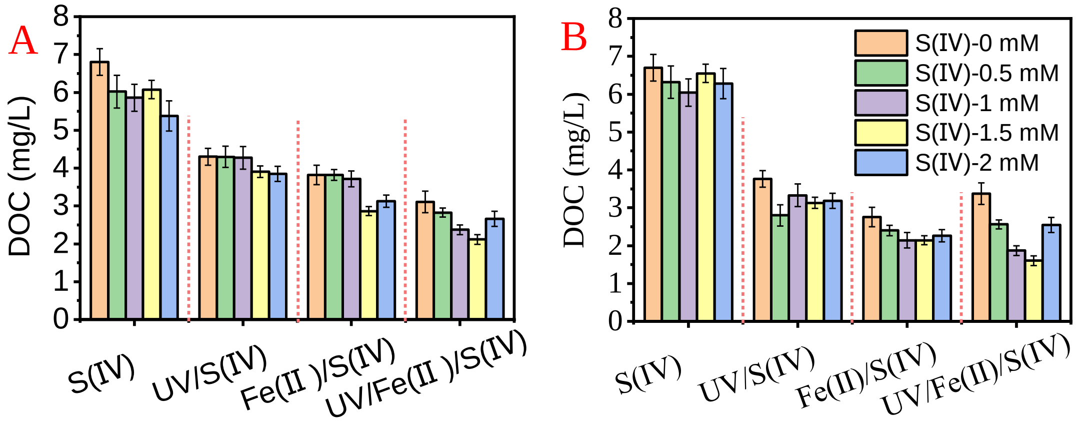

Figure S2. Permeate water DOC in varying systems treating (A) EOM solution and (B) ALW.

Normalized flux of EOM filtration in different pretreatment system with varying S(IV) dosages.

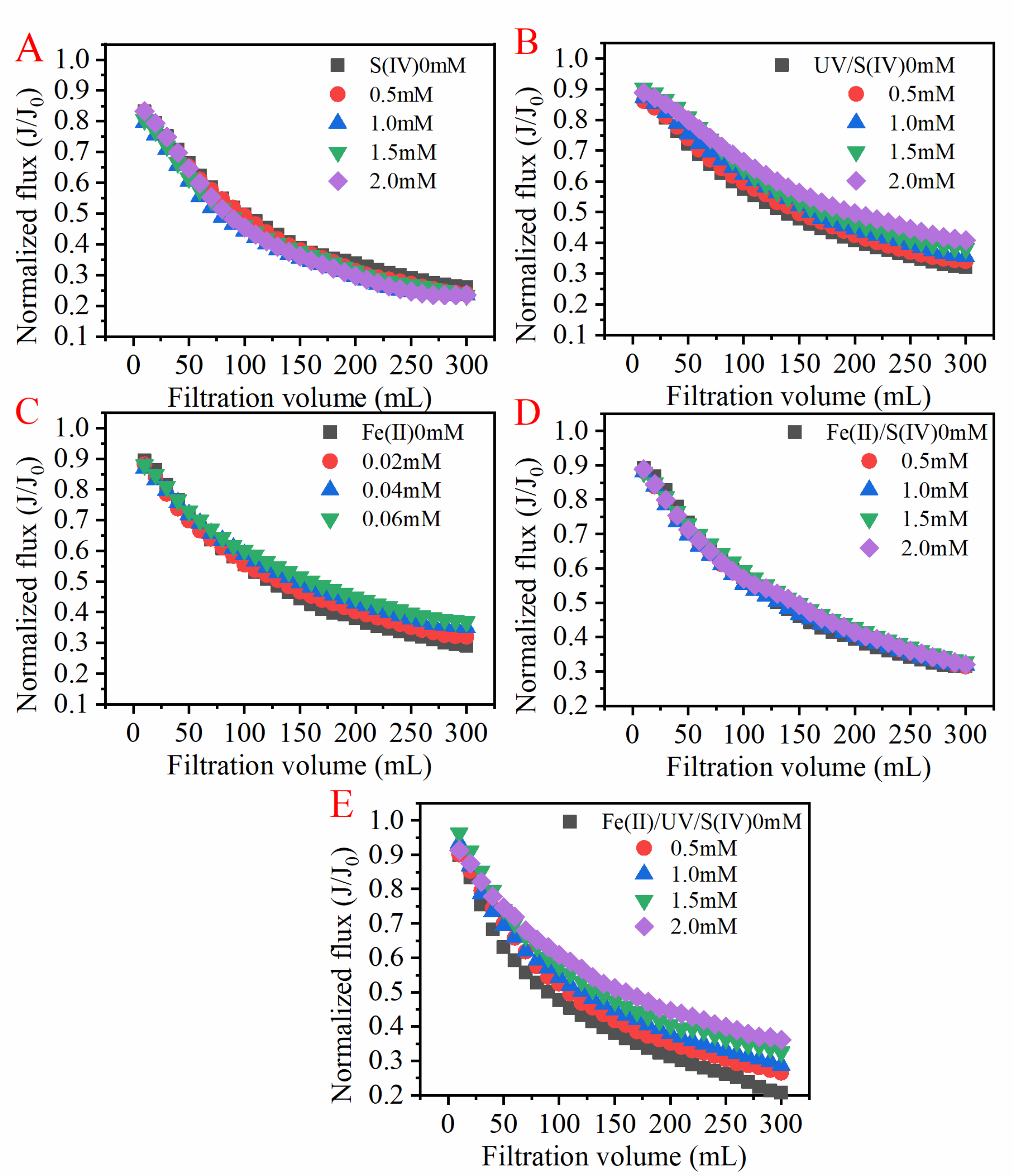

Figure S3. Membrane flux decline with varying S(IV) dosages in different pretreatments treating EOM (A) stand-alone S(IV) ALW, (B) UV/S(IV), (C) stand-alone Fe(II), (D) Fe(II)/S(IV) and (E) UV/Fe(II)/S(IV).

Membrane fouling resistance of EOM filtration in different pretreatment system with varying S(IV) dosages

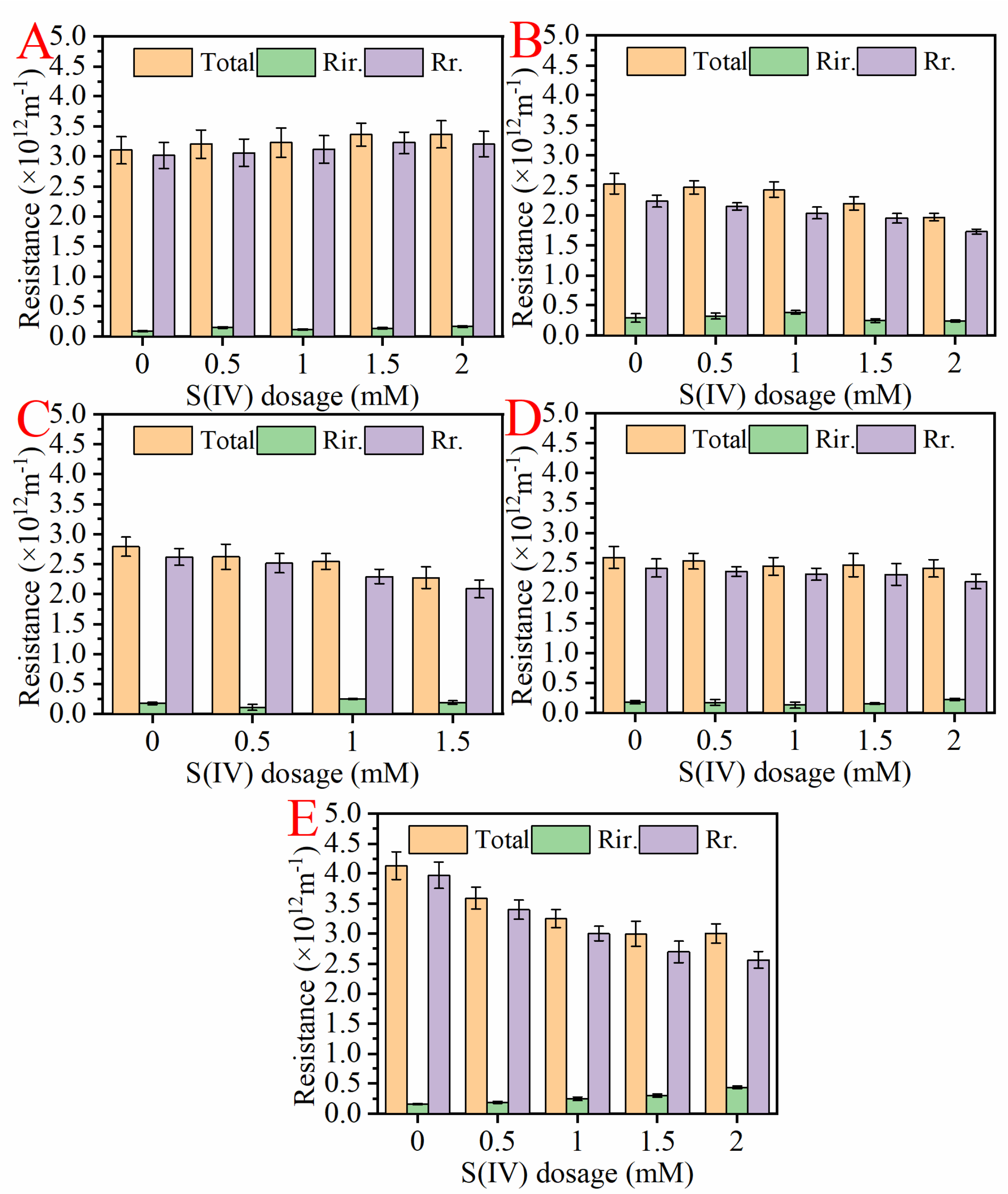

Figure S4. Membrane fouling resistance with varying S(IV) dosages in different systems treating EOM (A) stand-alone S(IV) ALW, (B) UV/S(IV), (C) stand-alone Fe(II), (D) Fe(II)/S(IV) and (E) UV/Fe(II)/S(IV).

Specific flux of ALW filtration in different pretreatment system with varying S(IV) dosages

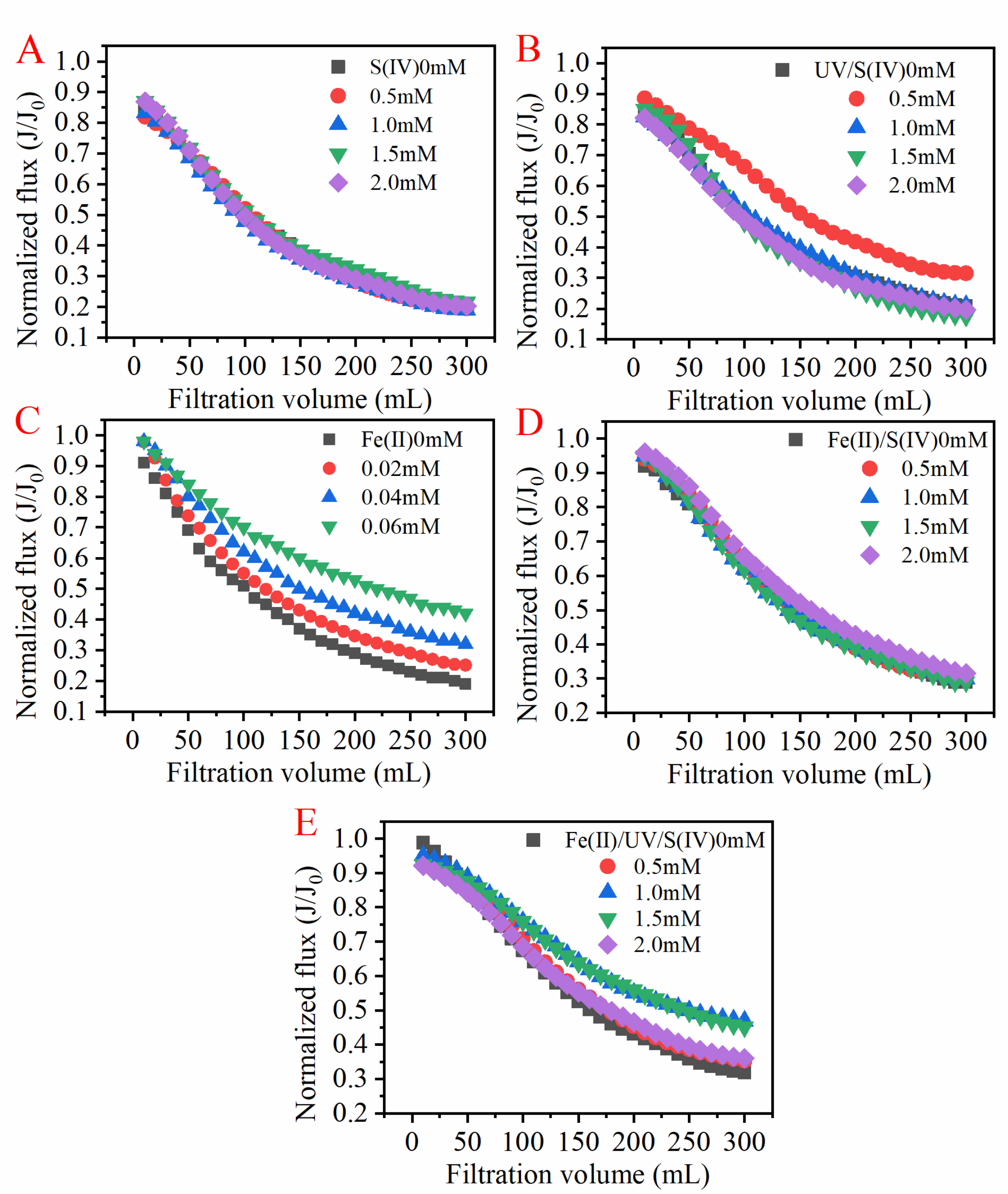

Figure S5. Membrane flux variation with varying S(IV) dosages in different systems treating ALW (A) stand-alone S(IV) ALW, (B) UV/S(IV), (C) stand-alone Fe(II), (D) Fe(II)/S(IV) and (E) UV/Fe(II)/S(IV).

Membrane fouling resistance of ALW filtration in different pretreatment system with varying S(IV) dosages

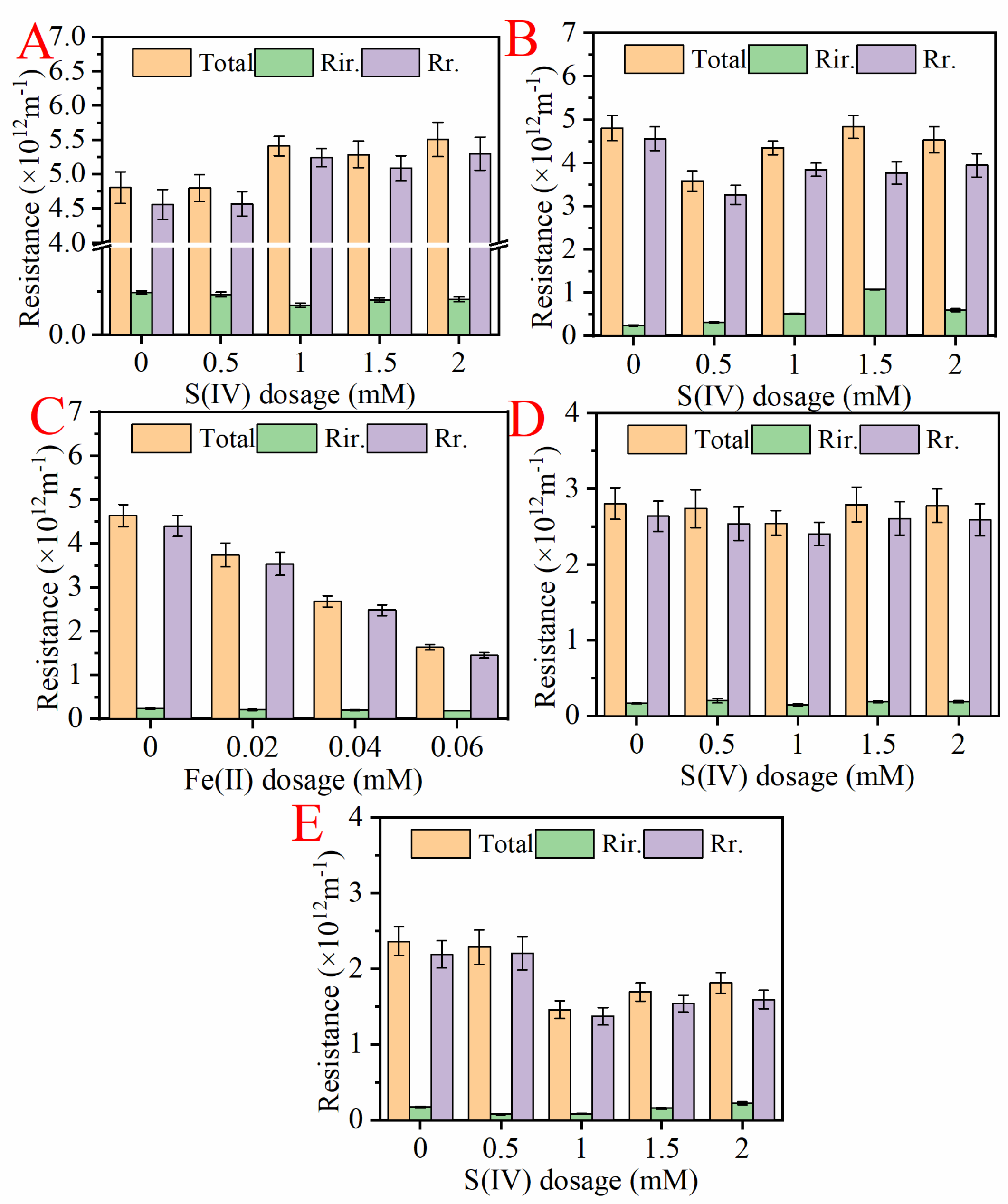

Figure S6. Membrane flux variation with varying S(IV) dosages in different systems treating ALW (A) stand-alone S(IV) ALW, (B) UV/S(IV), (C) stand-alone Fe(II), (D) Fe(II)/S(IV) and (E) UV/Fe(II)/S(IV).
